# Efficacy and safety of vildagliptin, sitagliptin, and linagliptin as add-on therapy in Chinese patients with T2DM inadequately controlled with dual combination of insulin and traditional oral hypoglycemic agent

**DOI:** 10.1186/s13098-015-0087-3

**Published:** 2015-10-19

**Authors:** Yun-Zhao Tang, Gang Wang, Zhen-Huan Jiang, Tian-Tian Yan, Yi-Jun Chen, Min Yang, Ling-Ling Meng, Yan-Juan Zhu, Chen-Guang Li, Zhu Li, Ping Yu, Chang-Lin Ni

**Affiliations:** Key Laboratory of Hormones and Development (Ministry of Health), Metabolic Diseases Hospital and Tianjin Institute of Endocrinology, Tianjin Medical University, Tongan Road 66, Heping District, Tianjin, 300070 China

**Keywords:** Add-on therapy to insulin, Type 2 diabetes mellitus, DPP-4 inhibitors, Glycemic control

## Abstract

**Objective:**

We aimed to evaluate the efficacy and safety of the three dipeptidyl peptidase 4 (DPP-4) inhibitors (vildagliptin, sitagliptin, and linagliptin) as add-on therapy in Chinese patients with type 2 diabetes mellitus (T2DM)inadequately controlled on dual combination of insulin and metformin or acarbose.

**Methods:**

A total of 535 T2DM patients who failed to achieve glycemic control with insulin and a traditional oral hypoglycemic agent were randomized to receive vildagliptin, sitagliptin, or linagliptin. Body mass index, glycosylated hemoglobin (HbA1c), fasting and postprandial plasma glucose (FPG and PPG), insulin dose, and adverse events were evaluated during the study.

**Results:**

The baseline HbA1c was 9.59 ± 1.84 % (vildagliptin group), 9.22 ± 1.60 % (sitagliptin group), and 9.58 ± 1.80 % (linagliptin group). At week 12 it was 8.16 ± 1.29 % (vildagliptin), 8.56 ± 1.96 % (linagliptin), and 8.26 ± 1.10 % (sitagliptin). The changes in HbA1c from baseline were −1.33 ± 0.11 % (vildagliptin), −0.84 ± 0.08 % (sitagliptin) and −0.81 ± 0.08 % (linagliptin), the vildagliptin group had the greatest reduction in HbA1c (*P* < 0.05). The proportions of patients that reached target HbA1c were 66.27 % (vildagliptin), 52.73 % (sitagliptin), and 55.49 % (linagliptin), the vildagliptin group had the highest one (*P* < 0.05). The baseline FPG and PPG values in the three groups were at the same level. At week 12, mean FPG levels in the vildagliptin (7.31 ± 1.50 mmol/L) and linagliptin (6.90 ± 1.55 mmol/L) groups were significantly lower than in the sitagliptin group (8.02 ± 4.48 mmol/L; *P* < 0.05); the linagliptin group had the lowest mean PPG followed by the vildagliptin group which was also significant lower (P = 0.000) than the sitagliptin group. Additionally, the required insulin dosage in the vildagliptin group was the lowest among the groups at weeks 6 and 12. Only mild AEs were reported during the study.

**Conclusion:**

The three DPP-4 inhibitors appear to be effective and safe as add-on therapy for T2DM patients on dual combination of insulin and a traditional OHA. Vildagliptin was more effective in decreasing insulin requirement and achieving glycemic control when compared to the other two.

## Background

Type 2 diabetes mellitus (T2DM) affects over 300 million people worldwide [1]. The global prevalence of T2DM was estimated to be 9 % among adults aged over 18 years in 2014 [1]. Excluding accidents, diabetes is the fifth cause of death for women and the fourth for men in the USA [2]. In China, the total diabetes prevalence was 9.7 % (92.4 million adults) according to the China National Diabetes and Metabolic Disorders Study between 2007 and 2008, while the prevalence of prediabetes was estimated to be 15.5 % (148 million adults) [3]. The increasing prevalence of diabetes has followed rapid economic growth, increases in life expectancy, and changes in lifestyle [3]. Inadequate control of blood glucose in patients correlates with a higher risk for diabetes-related micro and macrovascular complications [4, 5]. The management of diabetes aims at improving glycemic control to reduce the onset of complications [6]. Glycemic control is typically measured as reductions in glycosylated hemoglobin (HbA1c).

T2DM is a progressive disease that often requires a combination of antidiabetic drugs with different mechanisms of action to achieve glycemic targets over time [7]. Dipeptidyl peptidase 4 (DPP-4) inhibitors have become a useful class of oral hypoglycemic agents (OHA) for the treatment of T2DM since 2006. DPP-4 is a transmembrane glycoprotein located on the surface of most cell types, and its multiple effects may be associated with immune regulation, cell apoptosis, and signal transduction [8]. The clinically relevant action of DPP-4 is the degradation of endogenous glucagon-like peptide 1 (GLP-1). Additionally, DPP-4 inhibitors enhance insulin secretion in a glucose-dependent manner [9].

Several clinical practice guidelines recommend a stepwise treatment pathway for diabetes. Diet control and lifestyle intervention are considered the cornerstones for treatment of DM according to these guidelines. However, dietary and lifestyle changes are difficult to implement and maintain on a large scale. Given the progressive nature of T2DM, long-term glycemic control is difficult to achieve with a single agent, thus often requiring the addition of further agents [10]. The American Diabetes Association/European Association for the Study of Diabetes position statement and the American Association of Clinical Endocrinologists/American College of Endocrinology algorithm suggest the use of DPP-4 inhibitors as a second option when metformin fails [11]. Addition of a DPP-4 enzyme inhibitor to metformin treatment is beneficial owing to the complementary mechanisms of action of both drugs [11–14]. Nonetheless, the role of those new drugs in the treatment of T2DM is still debated [15]. A few clinical studies have reported the effect of DPP-4 inhibitors as an add-on therapy to insulin [16–18]. The efficacy in achieving glycemic control and weight-sparing effects of DPP-4 inhibitors have been stated in both systematic reviews and meta-analyses in comparison with placebo and other antidiabetic medications [19–21]. However, their safety and the efficacy when combined with traditional anti-hyperglycemic therapy remain inconclusive. Therefore, we conducted this study to evaluate the efficacy and safety of DPP-4 inhibitor vildagliptin, sitagliptin, or linagliptin as add-on therapy for T2DM patients inadequately controlled with dual combination of insulin and metformin or acarbose.

## Methods

### Subjects

Patients were recruited from the Metabolic Diseases Hospital of Tianjin Medical University between January 2013 and January 2015. Enrolled male or female patients met the following criteria: diagnosis of T2DM; age >18 years; HbA1c levels >7.0 %; body mass index (BMI) between 22 and 45 kg/m^2^ at visit 1 (week −4); current treatment with insulin at a stable dose of 20–80 U daily and a traditional OHA (metformin 750–1000 mg daily or acarbose 100–300 mg daily) for at least 12 weeks before screening.

Patients who met the following criteria were excluded from the study: pregnant or lactating women; diagnosis of type 1 diabetes mellitus, diabetes secondary to pancreatic injury or other types of secondary diabetes; acute metabolic diabetic complications, such as ketoacidosis or hyperosmolar state (coma) within the 3 months prior to enrollment; myocardial infarction, unstable angina or coronary artery bypass surgery within 6 months prior to enrollment; congestive heart failure (NYHA III-IV grade); history of liver disease such as cirrhosis, hepatitis B, or hepatitis C (except carriers), or alanine transaminase (ALT), aspartate aminotransferase (AST) greater than two times the upper limit of normal (ULN) or total bilirubin greater than two times the ULN; history of kidney disease or clinical diagnosis of renal insufficiency indicated by serum creatinine ≥132 μmol/L (≥1.5 mg/dL) in male patients, and ≥123 μmol/L (≥1.4 mg/dL) in female patients; thyroid-stimulating hormone beyond the normal range; and history of acute and chronic pancreatitis.

Written informed consent was obtained from each patient prior to enrollment. This study design was approved by the local ethics committee and review board and was in accordance with the Declaration of Helsinki.

### Study design

**Fig. 1:**
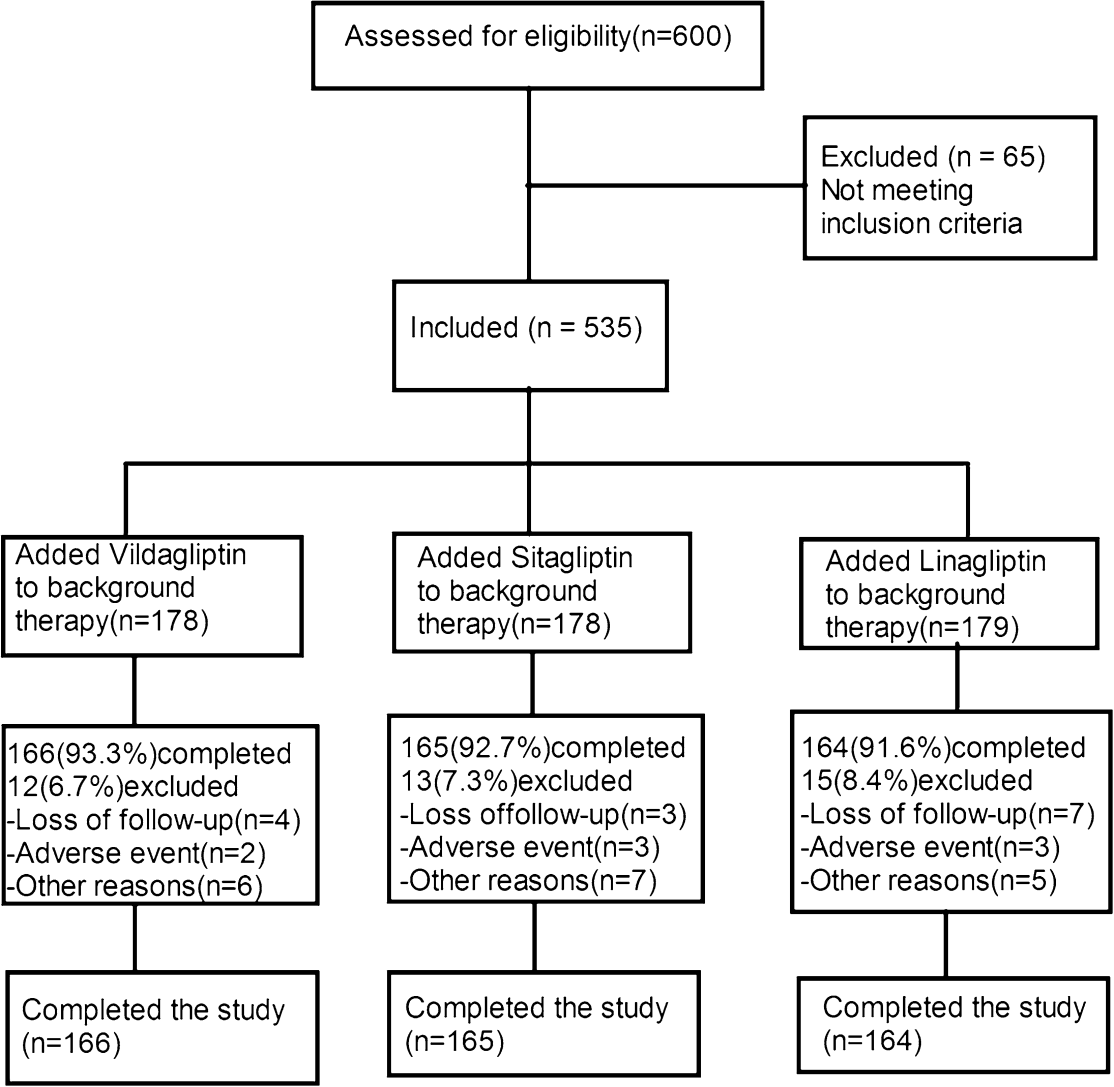
Flow diagram of the patient recruitment process

This was a 12-week, randomized, open-label, parallel clinical study. The flow diagram of the study design is presented in Fig. 1. Patients with T2DM who met the inclusion criteria were screened for eligibility at Visit 1 (week −4) and were randomized (1:1:1) at Visit 2 (week 0, baseline) to receive vildagliptin 50 mg bid, sitagliptin 100 mg qd, or linagliptin 5 mg qd for 12 weeks. Patients were required to maintain their individual eating and exercise habits during the study, and follow all the study requirements as well. At and after the screening visit, baseline laboratory and clinical data were evaluated in every case. The DPP-4 inhibitor was administered as an add-on therapy to the background OHA maintained throughout the study. The patients were followed up every 2 weeks in the outpatient department. Dose adjustments of insulin or analogs were performed by the treating physicians according to the level of the blood glucose at each visit.

### Study assessments and endpoints

The primary study endpoint was change in HbA1c and the proportion of patients that reached the target HbA1c level (<7.0 %) [22] from baseline to week 12. Secondary efficacy assessments included changes in FPG, PPG, and the insulin dose from baseline to endpoint (week 12). Safety assessments included recording and monitoring of treatment-emergent adverse events (AEs); biochemistry and hematology laboratory test results; electrocardiogram findings and vital signs. Hypoglycemia was defined as symptoms suggestive of hypoglycemia with a self-monitored plasma glucose measurement <3.1 mmol/L. Severe hypoglycemia was defined as an episode requiring external assistance or hospitalization with or without a plasma glucose measurement <3.1 mmol/L.

### Statistical analysis

All the measurement data were expressed as mean ± standard deviation. One-way analysis of variance was used to compare the differences in clinical characteristics between the three groups at baseline and after treatment. Fisher’s least significant difference test was adopted for multiple comparisons. Chi square test was performed for analysis of differences in the frequency distributions. The data were analyzed by SPSS 18.0 for Windows (SPSS Inc., Chicago, IL, USA). *P* < 0.05 was considered statistically significant.

## Results

### Patient Disposition and Baseline Characteristics

The disposition of patients from screening to study endpoint is depicted in Fig. 1. Of total 600 patients screened, 535 were randomized to receive vildagliptin (n = 178), sitagliptin (n = 178), and linagliptin (n = 179) respectively. 166 of 178 (93.3 %) patients in the vildagliptin group, 165 of 178 (92.7 %) in the sitagliptin group, and 164 of 179 (91.6 %) in the linagliptin group completed the study. Discontinuation in the three groups was mainly due to loss to follow-up, adverse events (mainly hypoglycemia), or other reasons.

The baseline characteristics of the randomized patients are presented in Table 1. The three groups were well balanced at baseline in terms of gender, age, BMI, disease duration, insulin dose and background therapy (insulin and OHA). All biochemistry indexes were matched between groups except for blood urea nitrogen (BUN).

**Table 1:** Baseline characteristics of the participants

Variables	Vildagliptin (n = 166)	Sitagliptin (n = 165)	Linagliptin (n = 164)	For χ^2^ values	*P* values
Male, n (%)	92 (55.4)	104 (63.0)	104 (63.4)	2.817	0.245
Age (years)	56.20 ± 10.37	56.04 ± 13.80	54.66 ± 10.85	0.851	0.428
BMI (kg/cm^2^)	26.49 ± 6.44	26.51 ± 4.29	26.82 ± 4.80	0.198	0.821
Disease duration (years)	7.78 ± 0.58	8.50 ± 0.62	7.51 ± 0.53	0.779	0.459
SBP (mmHg)	131.63 ± 16.41	130.92 ± 15.32	131.53 ± 18.71	0.085	0.918
DBP (mmHg)	79.85 ± 8.92	80.33 ± 7.93	81.38 ± 11.02	1.122	0.326
ALT (IU/L)	23.98 ± 13.83	23.87 ± 14.56	27.34 ± 16.70	2.397	0.092
AST (IU/L)	20.51 ± 9.36	22.75 ± 10.64	22.01 ± 10.98	1.898	0.151
BUN (mmol/L)	5.93 ± 1.79	6.32 ± 2.58	5.49 ± 1.35	5.639	0.004
Cr (umol/L)	66.08 ± 45.22	69.09 ± 23.88	64.08 ± 13.88	0.880	0.416
TG (mmol/L)	2.14 ± 2.01	2.29 ± 1.82	2.26 ± 1.72	0.286	0.751
TC (mmol/L)	5.16 ± 1.24	5.30 ± 1.36	5.09 ± 1.04	1.017	0.363
HbA1c (%)	9.59 ± 1.84	9.22 ± 1.60	9.58 ± 1.80	2.345	0.097
Insulin dose (U)	33.15 ± 1.00	36.02 ± 1.06	33.43 ± 1.23	2.121	0.121
Insulin therapy, n (%)					
Premixed human insulin	65 (39.15)	81 (49.00)	64 (39.02)	7.270	0.122
Premixed insulin analogs	42 (25.30)	44 (26.67)	41 (25.00)		
Basal insulin	59 (35.54)	40 (25.32)	59 (35.98)		
Background OHA, n (%)					
Metformin	80 (48.2)	93 (56.4)	90 (54.9)	2.519	0.284
Acarbose	86 (51.8)	72 (43.6)	74 (45.1)		

Values are expressed as mean ± SD or n (%). BUN in the linagliptin group was lower than the other two groups (*P* < 0.05)

*ALT* alanine transaminase, *AST* aspartate transaminase, *BMI* body mass index, *BUN* blood urea nitrogen, *SBP* systolic blood pressure, *Cr* creatinine, *DBP* diastolic blood pressure, *OHA* oral hypoglycemic agent, *TC* total cholesterol, *TG* triglyceride

### Efficacy

**Fig. 2:**
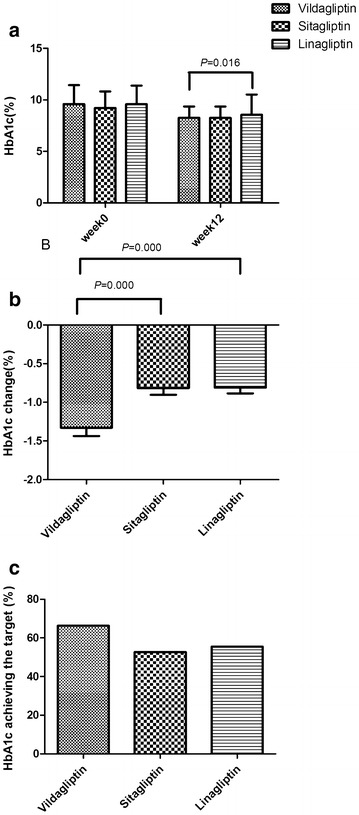
**a** HbA1c during the 12-week treatment with vidagliptin, sitagliptin, or linagliptin. There were no differences in HbA1c at baseline. Levels of HbA1c in the vildagliptin group were lower than in the linagliptin group at week 12 (P < 0.05). **b** HbA1c changes during the 12 weeks in the vildagliptin, sitagliptin, or linagliptin group. The change in HbA1c in the vildagliptin group was the greatest among the three groups (P < 0.05). **c** Proportion of patients. The proportion of patients achieving the target HbA1c levels in the vildagliptin group was the greatest among the three groups (P < 0.05)

At baseline HbA1c was 9.59 ± 1.84 % in the vildagliptin group, 9.22 ± 1.60 % in the sitagliptin group, and 9.58 ± 1.80 % in the linagliptin group, no differences was found between the three groups (P = 0.097). After 12 weeks of treatment, it was reduced to 8.16 ± 1.29 % (vildagliptin), 8.56 ± 1.96 % (linagliptin), and 8.26 ± 1.10 % (sitagliptin). Except that the vildagliptin group had a lower HbA1c value than the linagliptin group (P = 0.044), no significant differences in HbA1c were found between the groups (Fig. 2a).

The change in HbA1c from baseline was the most important end point of our study. As mentioned above all three groups had a decline in HbA1c but the vildagliptin group had the greatest one (−1.33 ± 0.11 %) (*P* = 0.000). The changes in HbA1c were −0.84 ± 0.08 and −0.81 ± 0.08 % in the sitagliptin and linagliptin groups, respectively (Fig. 2b).

66.27 % of patients in the vildagliptin group achieved target HbA1c level, whereas in sitagliptin group the proportion was 52.73 % and in the linagliptin group 55.49 %. The vildagliptin group had the highest proportion that reached target HbA1c among the three groups (P < 0.05) (Fig. 2c).

**Fig. 3:**
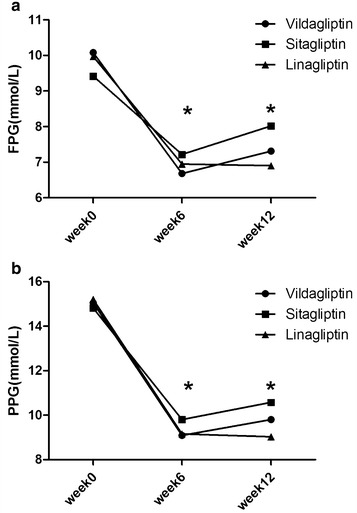
**a** Changes in FPG during the 12-week treatment with vildagliptin, sitagliptin, or linagliptin. The FPG levels in the three groups showed no difference at week 0. The FPG in the vildagliptin group was the lowest at week 6 (*P < 0.05). The FPG levels in the vildagliptin and linagliptin groups were lower than in the sitagliptin group at week 12 (*P < 0.05). **b** Changes in PPG during the 12 weeks in the vildagliptin, sitagliptin, or linagliptin group. The PPG levels were not significantly different between the groups. The PPG levels in the vildagliptin and linagliptin groups were lower than in the sitagliptin group at week 6 (*P < 0.05). The PPG in the linagliptin group was the lowest one, and the PPG in the vildagliptin group was lower than in the sitagliptin group at week 12 (*P < 0.05)

Mean FPG values during the 12 weeks of treatment are presented in Fig. 3a. The baseline values in the three groups were at the same level. At week 6, they were 6.68 ± 1.03 mmol/L in the vildagliptin group, 7.22 ± 1.47 mmol/L in the sitagliptin group, and 6.95 ± 1.27 mmol/L in the linagliptin group. All three groups revealed a decline in FPG when compared with their baseline levels, but it was more significant in the vildagliptin arm than in the other two groups (P = 0.001). At week 12, mean FPG levels were 7.31 ± 1.50 mmol/L in the vildagliptin group and 6.90 ± 1.55 mmol/L in the linagliptin group, significantly lower when compared with the sitagliptin group (8.02 ± 4.48 mmol/L; P = 0.002).

The mean PPG values during the 12 weeks of the study are depicted in Fig. 3b. The values at baseline were 15.05 ± 4.02 mmol/L in the vildagliptin group, 14.82 ± 3.58 mmol/L in the sitagliptin group, and 15.21 ± 3.78 mmol/L in the linagliptin group. No differences were found between the three groups (*P* = 0.653). All three groups revealed a reduction in PPG after treatment, but to a slightly different degree. At week 6, the mean PPG was 9.09 ± 1.83 mmol/L (vildagliptin), 9.16 ± 2.24 mmol/L (linagliptin) and 9.80 ± 2.23 mmol/L (sitagliptin). The value was significantly lower in the vildagliptin group and linagliptin group than in the sitagliptin group (P = 0.004). At week 12, the value was 9.03 ± 2.53 mmol/L (linagliptin), 9.80 ± 2.22 mmol/L (vildagliptin) and 10.58 ± 2.64 mmol/L (sitagliptin). The linagliptin group had the lowest mean PPG followed by the vildagliptin group. Although the value in the vildagliptin group was not as low as in the linagliptin group, it was significantly lower (P = 0.000) than that in the sitagliptin group.

**Fig. 4:**
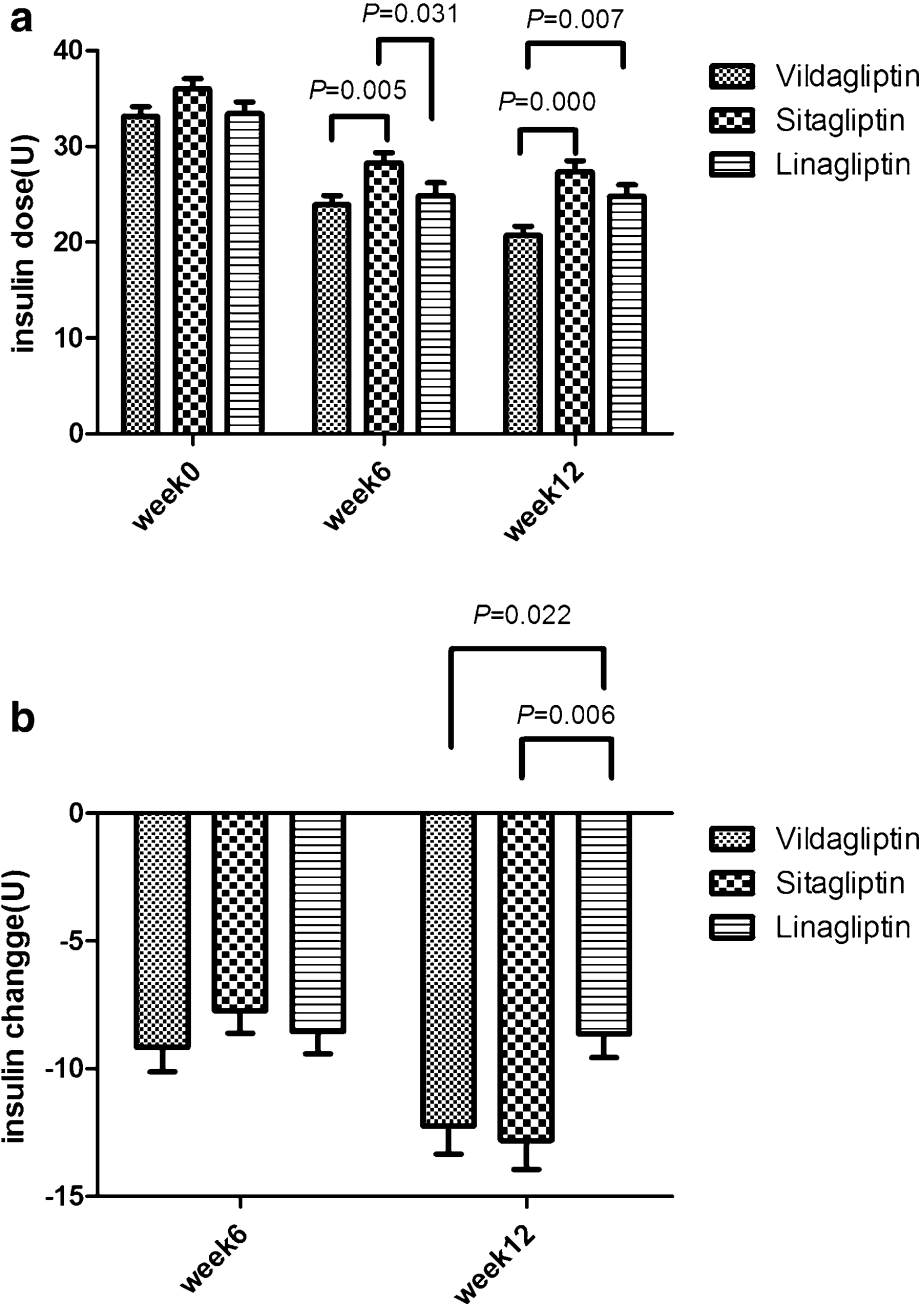
**a** Insulin doses during the 12-week treatment with vildagliptin, sitagliptin, or linagliptin. The insulin doses at week 0 were not significantly different. The insulin dose in the vildagliptin group was lower than in the sitagliptin group at week 6. The insulin dose in the linagliptin group was lower than in the sitagliptin group at week 6. The mean insulin dose in the vildagliptin group was much lower than in the other two groups at week 12 (P < 0.05). **b** Insulin changes during the 12-week treatment with vildagliptin, sitagliptin, or linagliptin. There were no significant differences between the three groups at week 6. The insulin changes in the vildagliptin and sitagliptin groups were much more pronounced than in the linagliptin group (P < 0.05). *FPG* fasting plasma glucose, *HbA1c* glycosylated hemoglobin, *PPG* postprandial plasma glucose

A decline in mean insulin dose was noticed in the three groups over the 12-week study period. At baseline, the dose was 33.15 ± 12.89 U in the vildagliptin group, 36.02 ± 13.09 U in the sitagliptin group, and 33.43 ± 15.76 U in the linagliptin group (P = 0.121). At week 6 the dose was 23.92 ± 0.96 U (vildagliptin), 24.89 ± 1.28 U (linagliptin) and 28.29 ± 1.06 U (sitagliptin). The vildagliptin group had the lowest insulin dose. The dose in the linagliptin group, though not as low as in the vildagliptin group, was lower than in the sitagliptin group (P = 0.014). At week 12 the dose was 20.71 ± 12.36 U (vildagliptin), 27.34 ± 13.46 U (sitagliptin) and 24.81 ± 15.08 U (linagliptin). Comparison between groups showed that the mean insulin dose was much lower in the vildagliptin group than in the other two groups (*P* = 0.000) (Fig. 4a).

In the present study change in insulin dose was an important variable which demonstrated a downward trend in the three groups. At week 6, the change was −9.17 ± 0.95 U in the vildagliptin group, −7.73 ± 0.86 U in the sitagliptin group, and −8.85 ± 0.88 U in the linagliptin group. No differences were found between the three groups. At week 12, however, the change was −12.24 ± 1.11 U (vildagliptin), −12.81 ± 1.13 U (sitagliptin) and −8.63 ± 0.93 U (linagliptin). The change was more pronounced in the vildagliptin group and sitagliptin group than in the linagliptin group (P = 0.013) (Fig. 4b).

We measured the participants’ BMI, blood pressure, and lipid profile during the 12-week follow-up. There were no changes in BMI and blood pressure. The total cholesterol (TC) and triglycerides (TG) in the three groups showed a downtrend when compared with the baseline, but the differences were not significant. In addition no differences in TC and TG at week 6 or 12 were found between the groups (data not shown).

### Safety

No severe AEs were reported in the three groups. All the AEs reported during the study were mild. The most commonly reported AEs were gastrointestinal AEs (14.46 % for vildagliptin, 11.52 % for sitagliptin, and 9.15 % for linagliptin). The other frequently reported AE was hypoglycemia (12.05 % for vildagliptin, 10.3 % for sitagliptin, and 7.29 % for linagliptin). There were very low incidences of renal and hepatic toxicity, infections, and chest discomfort. There was no significant difference between groups in terms of reported AEs (Table 2).

**Table 2:** Adverse events during the 12 weeks

Variables, n (%)	Vildagliptin (n = 166)	Sitagliptin (n = 165)	Linagliptin (n = 164)	χ^2^ values	*P* values
Hypoglycemia	20 (12.05)	17 (10.30)	13 (7.29)	1.554	0.460
Gastrointestinal adverse events	24 (14.46)	19 (11.52)	15 (9.15)	2.260	0.323
Renal and hepatic toxicity	6 (3.61)	5 (3.30)	2 (1.22)	2.008	0.366
Infections	10 (6.02)	8 (4.85)	12 (7.32)	0.881	0.644
Chest discomfort	8 (4.82)	11 (6.70)	11 (6.71)	0.676	0.713

## Discussion

The inability of monotherapy to maintain good glycemic control in T2DM as a result of progressive deterioration of β-cell function provides the rationale for the early use of combination therapy with different classes of drugs. For the same reason, insulin therapy is frequently required to achieve sufficient glycemic control. However, insulin therapy may lead to weight gain, increasing risk of hypoglycemia, edema, and some other side effects [23]. Considering all these benefits and harms, the chosen therapeutic regimen must be balanced to achieve glycemic control and decrease the dose of insulin needed.

This 12-week, randomized, open-label, parallel study evaluated the efficacy and safety of vildagliptin, sitagliptin, or linagliptin in Chinese patients with T2DM inadequately controlled on dual combination of insulin and a traditional OHA. All the groups achieved a better glycemic control compared with baseline both at weeks 6 and 12. The FPG values decreased in the three groups after treatment with vildagliptin, sitagliptin, or linagliptin but to a slightly different degree. At week 12, vildagliptin and linagliptin induced a significantly greater decrease in FPG than sitagliptin did. Similarly, vildagliptin and linagliptin induced significantly greater decline in PPG when compared with sitagliptin. At week 12, patients treated with linagliptin revealed the most remarkable decrease in the PPG levels, followed by vildagliptin. The three DPP-4 inhibitors showed excellent effect on glycemic control as add-on therapy in treating T2DM. Our results are in accordance with previous reports [16, 18, 24]. The present study showed vildagliptin, sitagliptin, and linagliptin help decrease FPG and PPG as add-on therapy to the background insulin treatment.

Our primary study end point was the change in HbA1c during the follow-up. At week 12 all three groups achieved significant change in HbA1c but the change in the vildagliptin arm was statistically greater than in the other two groups. Several previous reports demonstrated that vildagliptin, sitagliptin, and linagliptin are efficacious in decreasing HbA1c and enabled patients to reach glycemic control targets both as monotherapy and combination therapy [18, 19, 25–27]. Our study results are similar to those of previous studies in these regards. Moreover, as far as we know, this study is the first to compare vildagliptin, sitagliptin, and linagliptin as add-on therapy for T2DM patients with background insulin treatment. And what is more, our results indicate that vildagliptin is more effective in decreasing HbA1c.

It is generally acknowledged that high insulin doses might cause various unwanted effects during the treatment of T2DM. The present study extended the DPP-4 inhibitor treatment period to 12 weeks so that we were able to see the gradual decrease in blood glucose and the consequent decline in HbA1c. The gradual decline of blood glucose substantially decreased insulin requirement and resulted in considerable insulin dose reduction. Insulin doses in the vildagliptin group at weeks 6 and 12 were the lowest among the three groups. Additionally, the changes in insulin doses in the vildagliptin and sitagliptin groups were much more pronounced than in the linagliptin group.

The classical mechanism of the DPP-4 inhibitors to achieve glycemic control is based on the notion that these drugs increase the active levels of incretin hormones, GLP-1 and glucose-dependent insulinotropic polypeptide (GIP), and thereby improving pancreatic A- and B cell sensitivity to glucose [28]. A growing body of evidence both from clinical and preclinical findings supports the notion that DPP-4 inhibitors can ameliorate insulin resistance through several pathways [29, 30]. Thus, these findings might be potential explanations as to why adding DPP-4 inhibitors to background insulin therapy could considerably decrease the required dosage of insulin.

The safety of DPP-4 inhibitors as add-on therapy to background insulin treatment in T2DM patients was one of the relevant aspects being evaluated in the present study. The main AEs included gastrointestinal AEs, hypoglycemia, renal and hepatic toxicity, infections (including upper respiratory and urinary tract infections and nasopharyngitis), and chest discomfort. During this study, no pancreatitis was reported. The risk of hypoglycemia was similar in the three groups. As previously reported in meta-analyses, the rate of hypoglycemia was not higher in T2DM patients treated with the DPP-4 inhibitors, even in patients already undergoing treatment with insulin or a sulfonylurea [31, 32]. In accordance with our results, a meta-analysis reported that there was no increased incidence of gastrointestinal disorders in patients treated with DPP-4 inhibitors [33]. Moreover, the rates of other AEs in the present study were low and were not significantly different between the three groups.

The present study had certain limitations that need to be recognized. The study was performed in a relatively small number of patients at a single institution. Further studies are required to evaluate the long-term effects of DPP-4 inhibitors with or without insulin on glycemic control and insulin secretion in a larger number of patients. We did not recruit a group without DPP-4 inhibitors as controls, which is also a limitation to this study. Additionally, close pharmacovigilance monitoring plans are necessary to address the uncertainty regarding AEs of DPP-4 inhibitors, while their potential impact on cardiovascular outcomes will be clarified in the near future after the completion of more relevant long-term studies.

## Conclusions

Vildagliptin, sitagliptin, or linagliptin appear to be effective and safe as add-on treatment for T2DM patients inadequately controlled on dual combination of insulin and another OHA. This is the first study to evaluate the efficacy and safety of this treatment regimen in Chinese T2DM patients. The three DPP-4 inhibitors had similar efficacy in achieving glycemic control, but vildagliptin was much more efficacious in decreasing insulin requirement and achieving target HbA1c level. Therefore, we consider that vildagliptin or any of the other tested DPP-4 inhibitors could be added to combination therapy to reach the glycemic control in inadequately controlled patients.

## Abbreviations

ALT: alanine transaminase
AST: aspartate transaminase
BMI: body mass index
BUN: blood urea nitrogen
Cr: creatinine
DBP: diastolic blood pressure
FPG: fasting plasma glucose
GIP: glucose-dependent insulinotropic polypeptide
GLP-1: glucagon-like peptide-1
HbA1c: glycosylated hemoglobin
OHA: oral hypoglycemic agent
PPG: postprandial plasma glucose
SBP: systolic blood pressure
TC: total cholesterol
TG: triglyceride
ULN: upper limit of normal
